# Competency‐based training boosts dementia knowledge and skills in home care workers

**DOI:** 10.1002/alz.70323

**Published:** 2025-06-19

**Authors:** Jarmin Yeh, Matthew Beld, Brittney Pond, Melinda Neri, Andrea Garcia, Juliana Mata‐Pacheco, Juvenal Mauricio, Moraima Castanenda, Corinne Eldridge, Suzanna Martinez

**Affiliations:** ^1^ Institute for Health & Aging University of California San Francisco California USA; ^2^ Department of Social & Behavioral Sciences University of California San Francisco California USA; ^3^ Center for Caregiver Advancement Los Angeles California USA; ^4^ Department of Biostatistics and Epidemiology University of California San Francisco California USA; ^5^ Present address: Institute for Health & Aging University of California, San Francisco San Francisco CA USA

**Keywords:** caregiving, direct care workforce, home care, training, workforce challenges

## Abstract

**INTRODUCTION:**

The rising prevalence of Alzheimer's disease and related dementias (ADRD) in California's aging population necessitates a well‐trained dementia care workforce.

**METHODS:**

This study evaluated a multi‐week, competency‐based, online training for caregivers in California's Medicaid‐funded In‐Home Supportive Services (IHSS) program. Using a quasi‐experimental design, we assessed caregivers’ dementia knowledge, self‐efficacy, distress, depression, and care recipients’ healthcare use before and after the intervention.

**RESULTS:**

The training significantly improved caregivers’ dementia knowledge and self‐efficacy but did not reduce caregivers’ distress and depression, nor decrease care recipients’ emergency room visits and hospitalizations.

**DISCUSSION:**

The findings highlight the value of specialized dementia training in enhancing caregiver knowledge and skills, which could be implemented outside California. Clinical implications include bolstering caregiver well‐being to improve the quality of their support of care recipients with cognitive impairment. Policy implications include expanding access to training programs and bolstering workforce development initiatives that improve caregiver and care recipient outcomes.

**Highlights:**

- Multiweek online training improved home care workers’ dementia caregiving skills.
- Home care workers’ dementia knowledge improved significantly post‐training.
- Self‐efficacy to manage care recipients’ dementia symptoms improved significantly.

## 1 BACKGROUND

The increasing prevalence of Alzheimer's disease and related dementias (ADRD) highlights the critical role of direct care workers, including home care workers, in long‐term care for older adults.^1^, ^2^, ^3^, ^4^ These workers are essential to home and community‐based services, a sector facing unprecedented demand due to demographic shifts. In California, the home care workforce has rapidly expanded in response to an aging population and the rising prevalence of ADRD.^5^, ^6^ Despite a 483% increase from 138,800 to 796,890 workers between 2014 and 2023, the sector continues to face workforce shortages.^6^, ^7^ This growing yet invisible workforce remains undervalued, underutilized, and undertrained, despite being essential during the coronavirus disease 2019 (COVID‐19) pandemic by providing extended support during shelter‐in‐place and assisting care recipients with telehealth appointments.^8^, ^9^, ^10^ Issues such as caregiver burden, anxiety, and loneliness are prevalent but often overlooked in the broader healthcare context.^6^, ^11^, ^12^, ^13^

California's In‐Home Supportive Services (IHSS) program exemplifies the growing demand for home‐based care. This statewide Medicaid benefit program provides long‐term services and supports to older adults and adults with disabilities who require assistance to live safely in their homes. As of December 2023, approximately 656,417 paid caregivers served 750,537 people with low income, making IHSS the largest consumer‐directed personal care assistance program in the United States.^14^, ^15^ Consumer direction allows informed individuals to make choices about the services they receive, how they receive them, and by whom.

The IHSS program faces limitations in serving individuals with medical or cognitive conditions that may impair their ability to self‐direct care, increasing the risk of institutionalization.^5^ However, IHSS caregivers who spend intimate time with their care recipients are well‐positioned to observe changes in their care recipient's cognition, health, or behaviors to discuss it with them and with healthcare providers. Approximately 70% or more of the IHSS workforce are family caregivers. The remaining members are non‐family independent providers, who also play a vital role in ensuring the quality of life of their care recipients.^6^, ^14^, ^16^, ^17^, ^18^ These caregivers provide essential custodial care, assist with activities of daily living, and support their care recipients’ overall health and psychosocial well‐being, such as encouraging healthy behaviors, monitoring chronic conditions, and communicating with healthcare providers.^6^, ^14^, ^16^, ^19^, ^20^ Although caregivers play a crucial role in enhancing the quality of care and reducing healthcare utilization,^21^, ^22^ systemic drivers, including low wages and inconsistent access to quality dementia training programs across the state, perpetuate inequities in care for caregivers and care recipients alike.^6^, ^8^, ^23^, ^24^, ^25^

The development of educational interventions to foster home care workers’ dementia care capabilities has been a concern of researchers, yet further evaluation of these interventions is needed.^26^, ^27^, ^28^, ^29^, ^30^, ^31^, ^32^, ^33^ To address these gaps, we implemented the IHSS+ ADRD Training Project, a community‐based participatory research and program evaluation hybrid study that adapted an existing dementia curriculum for IHSS caregivers and assessed its real‐world effectiveness. The goal was to provide dementia education to 600 IHSS caregivers of care recipients with cognitive impairment in Alameda County, California.

**RESEARCH IN CONTEXT**

1. **Systematic review**: We conducted a review of dementia training interventions for home care workers, analyzing scientific literature from databases like PubMed and gray literature from industry reports, and a scoping review of intervention evaluations using scientific sources.
2. **Interpretation**: Our findings demonstrate that multi‐week online training can effectively enhance caregivers’ dementia knowledge and self‐efficacy. However, we observed limited changes in caregivers’ distress and depression, as well as care recipients’ healthcare service use. These results underscore the complex dynamics of home care, suggesting the need for more comprehensive support approaches.
3. **Future directions**: Future priorities should focus on strengthening clinical and policy support for home care workers' well‐being. Areas for investigation include enhanced study designs examining subgroup effects, longitudinal impacts, healthcare utilization patterns, and caregiver integration into care teams. Additional efforts should expand access to training across and beyond California and bolster workforce development policies, including compensation and benefits improvements.

**FIGURE 1:**
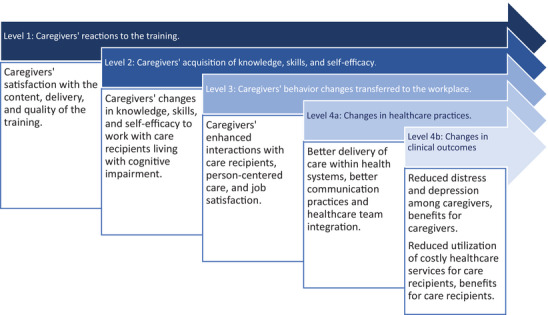
Impacts of the IHSS+ ADRD training project mapped to a modified version of the Kirkpatrick–Barr framework for health workforce education evaluation. ADRD, Alzheimer's disease and related dementias; IHSS, In‐Home Supportive Services.

We used a modified version of the Kirkpatrick–Barr framework for health workforce education evaluation to inform our analysis plan at four levels (Figure 1).^34^, ^35^, ^36^, ^37^, ^38^, ^39^ Level 1 assessed caregivers’ reactions to the content, delivery, and quality of the training. Level 2 evaluated caregivers’ acquisition of knowledge, skills, and self‐efficacy related to dementia caregiving. Level 3 evaluated proxy indicators of caregivers’ behavior changes transferred to the workplace, which was the home of their care recipient. Level 4a assessed proxy indicators of broader healthcare practice changes. Level 4b evaluated changes in clinical outcomes, including benefits for caregivers, such as changes in their distress and depression, and benefits to care recipients, such as their use of health system resources. We hypothesized that the training intervention would yield three specific outcomes: (1) improve caregivers’ ADRD knowledge and self‐efficacy, (2) decrease caregivers’ distress and depression, and (3) decrease care recipients’ emergency room visits and hospitalizations. Findings could help inform more targeted future interventions focusing on dementia training for the home care workforce.

## 2 METHODS

The IHSS+ ADRD Training Project was a 35‐h, 10‐week, competency‐based, instructor‐led, online training intervention delivered in English, Spanish, and Chinese (Cantonese and Mandarin) in Alameda County, California. We used a quasi‐experimental, longitudinal design to evaluate the effectiveness of the training intervention. The Institutional Review Board of the University of California, San Francisco approved the study (#19‐28395).

### 2.1 Intervention

To provide consistent and transparent reporting of the training intervention, we were informed by the guidelines for reporting evidence‐based practice educational interventions and teaching (GREET).^40^ We adapted a competency‐based dementia curriculum originally developed by the Geriatrics Workforce Enhancement Program at the University of California, Los Angeles, and the Center for Caregiver Advancement (CCA), a nonprofit founded by home care workers of Service Employees International Union (SEIU) Local 2015.^28^ In collaboration with the Alzheimer's Association, CCA refined the curriculum to include more in‐depth ADRD‐specific content on recognizing dementia symptoms, managing challenging behaviors (e.g., wandering, hallucinations, sundowning, etc.), and building communication skills. It also covered foundational IHSS caregiver responsibilities such as supporting activities of daily living, home safety, personal care, hygiene, medication management, and content on self‐care and reducing stress and burnout (Figure 2 ). To ensure local applicability, the curriculum was customized to incorporate resources and services offered by Alameda Alliance for Health (AAH), the primary Medicaid managed care organization in the county. This customization ensured the training was theoretically sound and pragmatically relevant to caregivers’ community context. Teaching and learning resources included workbooks, interactive individual and group exercises designed to encourage caregivers to reflect on how the content might be helpful in their role and setting, opportunities for discussion among caregivers to enhance social engagement through role‐playing, co‐learning, and social support; weekly assignments; and competency checks to reinforce knowledge and skills (Supporting Information, File). All print materials were created in English, Spanish, and traditional Chinese, with multilingual CCA instructors teaching in English, Spanish, Cantonese, and Mandarin to match caregivers’ language needs. The training consisted of approximately 3 h of content per session, each week, totaling 35 h over 10 weeks.

FIGURE 2IHSS+ ADRD training project weekly modules and learning objectives. ADRD, Alzheimer's disease and related dementias; IHSS, In‐Home Supportive Services.

**Figure:**
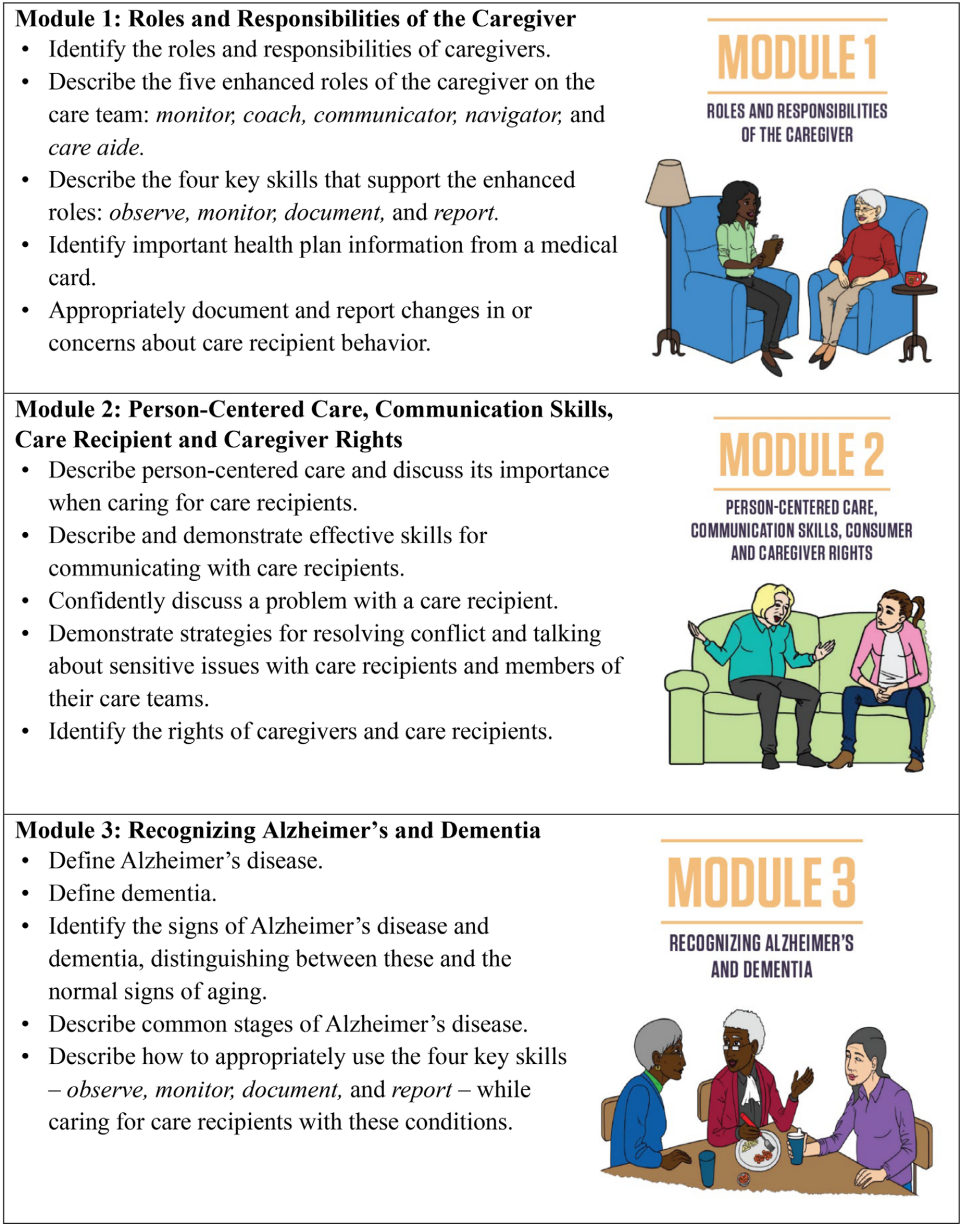

**Figure:**
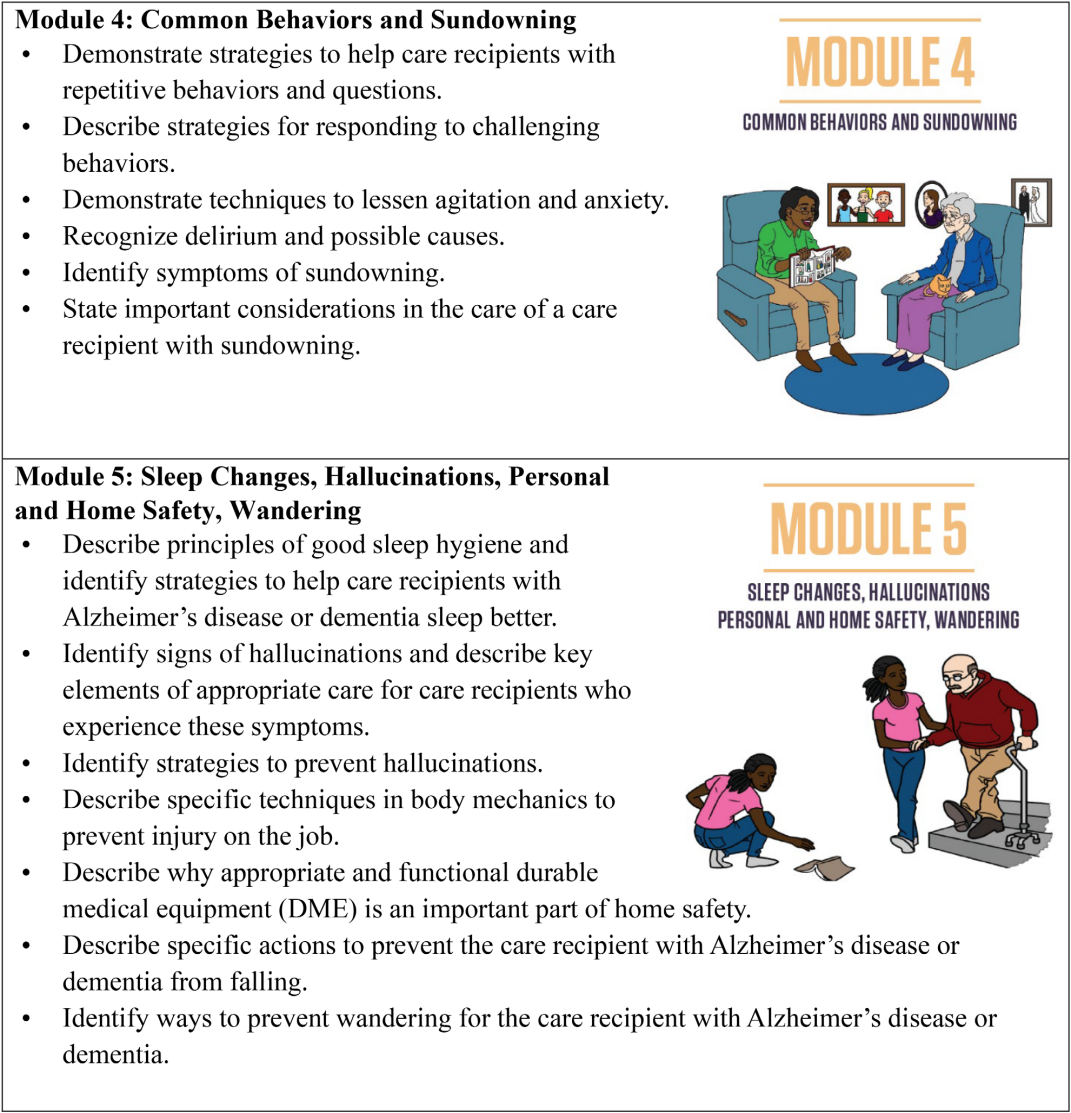

**Figure:**
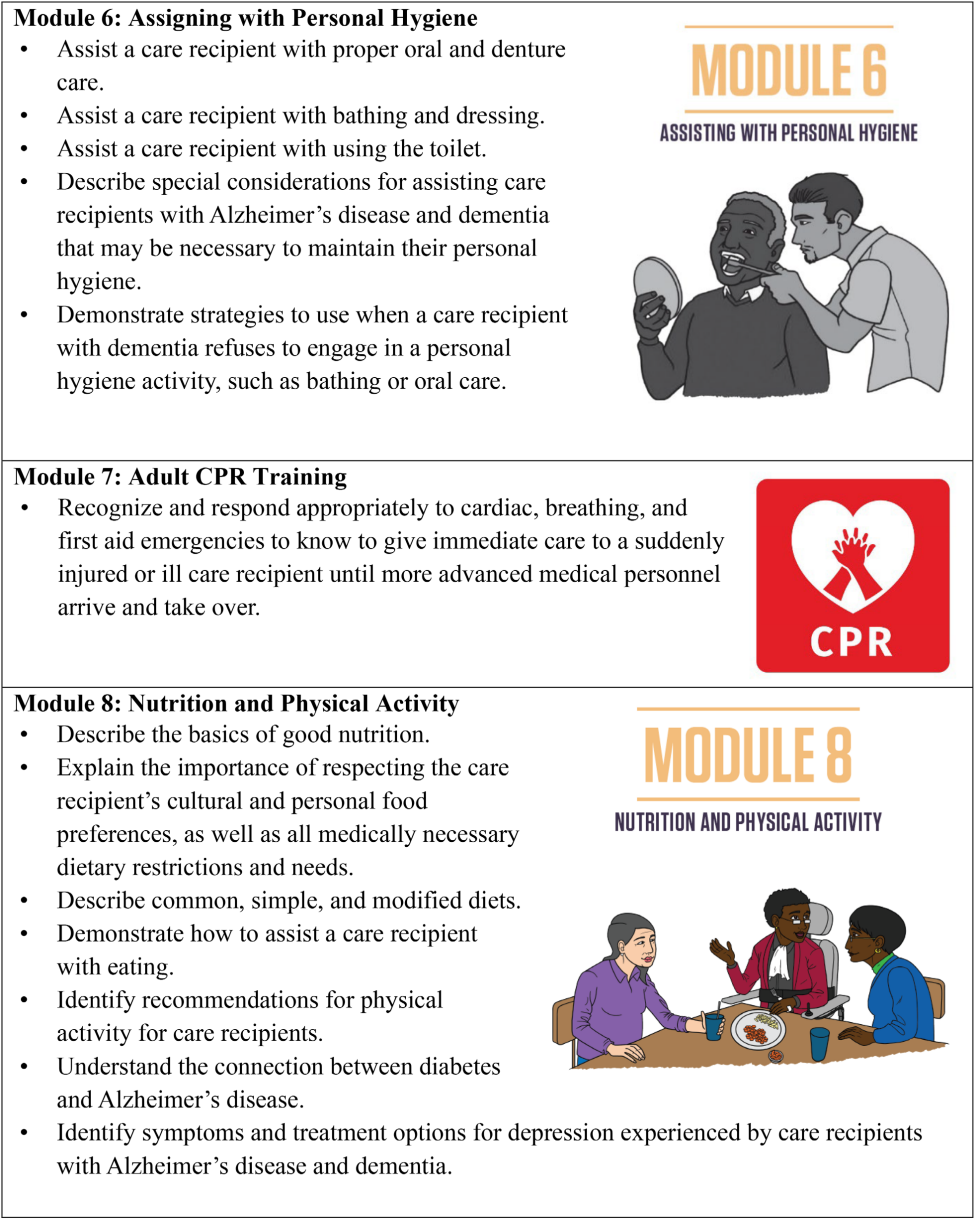

**Figure:**
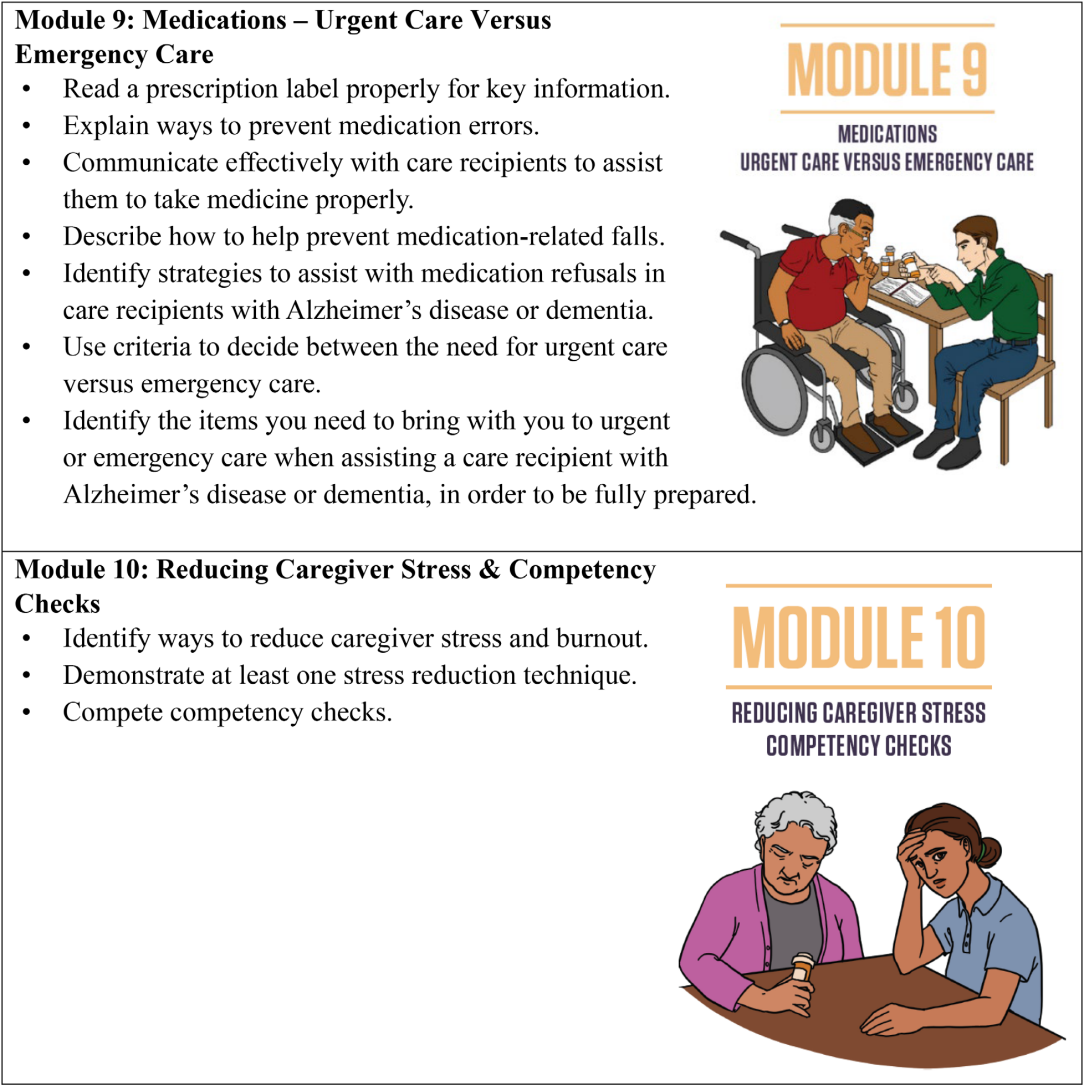

The training intervention was originally designed for in‐person instruction in a classroom setting. However, due to the COVID‐19 pandemic in March 2020, the IHSS+ ADRD Training Project was adapted to remote and virtual strategies. Research suggesting that online educational interventions improve the preparation of caregivers to provide high‐quality dementia care strengthened our conviction to make this transition.^41^ This shift enabled innovation, increased accessibility, and broader reach.

Adapting to virtual learning required a comprehensive overhaul to suit the needs of distance‐learning adults with varying levels of digital literacy. To improve attendance and engagement, CCA recruited caregivers through door‐knocking and in‐person home visits. The transition to remote recruitment and enrollment underscored caregivers’ difficulties with technology, such as unfamiliarity with texting, virtual consent forms, and Zoom, the video conferencing platform used for classes. CCA addressed these issues by providing follow‐up and reminder phone calls, video instructions, step‐by‐step guides, individualized phone support in various languages, and a new orientation module to help caregivers navigate Zoom. Additionally, CCA mailed Internet hotspots and iPads to caregivers who lacked reliable devices and connections, bridging the digital divide to bring education into their homes.

Caregivers were expected to attend all 10 weeks to graduate from the training. CCA instructors tracked attendance and followed up with caregivers who missed sessions via phone, text message, and email to schedule a makeup session. CCA instructors also arrived early to provide extra support with assignments or discuss lessons. Between September 2020 and December 2023, CCA delivered 31 online classes.

### 2.2 Study population

Caregivers had to meet the following inclusion criteria to be eligible for the training: age 18 or older; comfortable reading and speaking in English, Spanish, Cantonese, or Mandarin; and employed by an IHSS consumer who was age 50 or older. Care recipients had to be AAH health plan members and receive a score of 2 or greater on the Washington University Dementia Screening Test (AD8) based on the caregiver's assessment.^42^

The AD8 involves eight statements asking caregivers to indicate if there has been a change in the past several years caused by cognitive (thinking and memory) problems of their care recipient, with response options of “yes, a change,” “no change,” or “don't know.” The AD8 is sensitive in detecting early cognitive changes associated with common dementia‐related illnesses and has been validated in English, Spanish, and Chinese.^42^ We selected the AD8 because we could not identify the target care recipient population based on formal ADRD diagnoses from their patient records alone. Taking this approach was further justified by research indicating that approximately 59% of older adults with probable ADRD either do not receive or do not acknowledge a formal diagnosis.^43^, ^44^ Without official diagnostic information, we determined that the AD8 was a valuable proxy measure for identifying care recipients with potential cognitive impairment who also employed an IHSS caregiver. CCA obtained consent from both caregivers and care recipients to participate in the intervention. If care recipients could not consent, a designated power of attorney provided consent.

**FIGURE 3:**
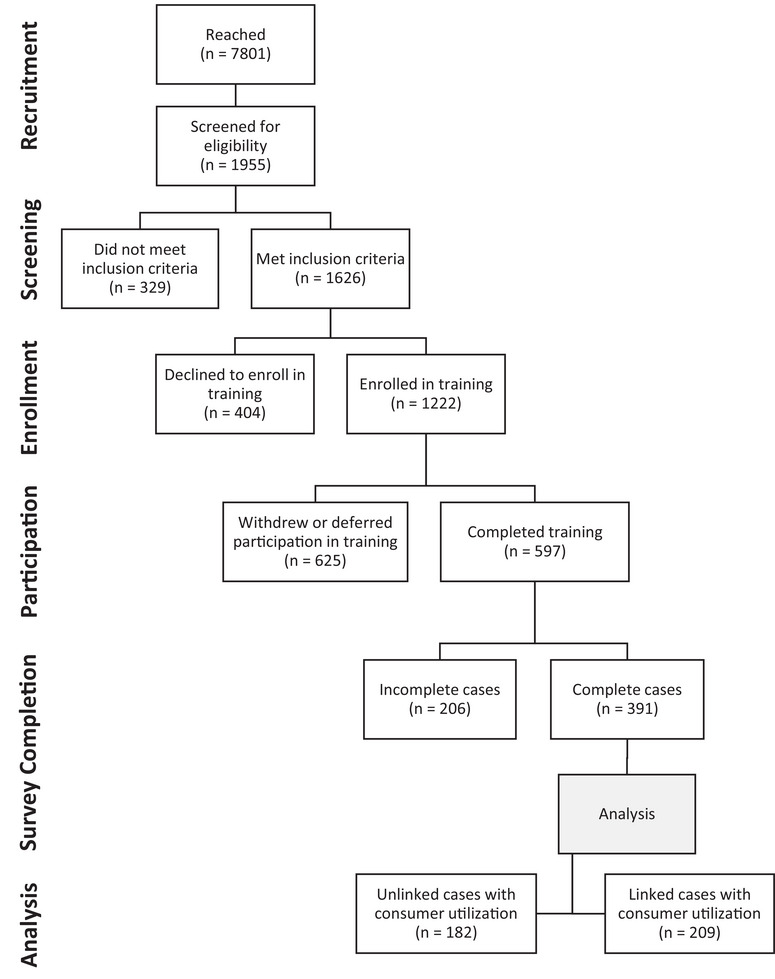
IHSS caregiver participant flow diagram. IHSS, In‐Home Supportive Services.

The target sample size of training 600 caregivers was based on a priori considerations of the likely geographic density of the target population in Alameda County. CCA led the recruitment of caregivers and care recipients using an ongoing, multi‐faceted campaign of phone calls, text messages, emails, and social media outreach (e.g., Facebook). The intervention was marketed as a free training program to help caregivers gain skills and knowledge to maximize the care they provide to their care recipient who may be showing signs of memory loss or cognitive decline. CCA reached 7801 people; 1955 were screened for eligibility if they expressed interest in the training; 1626 met inclusion criteria; 1222 were enrolled in the training intervention; 625 withdrew or deferred participation; and 597 caregivers completed all 10 weeks of the training intervention between September 2020 and December 2023. Figure 3 displays the participant's flow.

### 2.3 Evaluation design and data collection

Caregivers were asked to complete pre‐training, post‐training, and 3‐month follow‐up surveys. We designed the surveys to evaluate changes in job skills and knowledge, dementia knowledge, self‐efficacy, distress, depression, and training satisfaction. When appropriate, we obtained permission to use validated instruments and their Spanish‐ and Chinese‐translated versions from the researchers who developed them. For instruments and questions that needed translation, we used a professional language translation service. Biliterate CCA staff and instructors conducted back‐translations of these instruments and questions to verify the fit and acceptability of the translations for our target populations.^45^ The surveys included multiple‐choice questions with forced‐choice responses, questions where multiple items could be selected, and open‐ended questions where caregivers could write in responses.

Responses were collected through Qualtrics, a web‐based survey tool hosted by secure University servers. CCA distributed the surveys to caregivers during pre‐training, post‐training, and 3‐month follow‐up time periods. Paper mail‐and‐return surveys were available upon request. CCA received returned paper surveys and deidentified and digitized them by scanning and sharing them with the UCSF research team. A member of the UCSF research team entered the paper survey data into Qualtrics, which was spot‐checked for quality and accuracy. Unclear answers in the paper surveys were brought to the attention of the research team and collectively reviewed, discussed, and reconciled before being entered into Qualtrics. Most unclear answers were conservatively entered as “missing” information. We received 96 pre‐training, nine post‐training, and nine 3‐month follow‐up paper surveys. We received all other de‐identified surveys directly through Qualtrics. To encourage completion, CCA compensated caregivers with $100 for completing the training intervention and returning pre‐ and post‐training surveys, as well as an additional $200 for completing the 3‐month follow‐up survey.

### 2.4 Measures

#### 2.4.1 Level 1 measures—caregivers’ reactions to the training

We assessed pre‐training expectations by asking caregivers if they had taken any dementia training prior to participating in the IHSS+ ADRD Training Project and about what topics they would like to learn. We evaluated post‐training satisfaction by asking seven statements with response options on a five‐point scale (“strongly disagree” to “strongly agree”). We also assessed caregivers’ access to essential resources for participating in online training by asking four statements about technological and environmental resources with response options on a three‐point scale (“no access” to “consistent access”).

#### 2.4.2 Level 2 measures—caregivers’ acquisition of knowledge, skills, and self‐efficacy

We asked three questions aimed at capturing caregivers’ confidence in their professional skills, perceived competence in addressing care recipient needs, and asking for social support, with response options on a five‐point scale (“strongly disagree” to “strongly agree”). We evaluated job knowledge specific to IHSS caregiving (e.g., medication administration by IHSS caregivers under the supervision of a health professional) by asking five statements with response options of “true,” “false,” or “don't know.” We assessed knowledge about accessing various AAH resources by asking four statements with response options of “yes,” “no,” or “maybe.”

We evaluated foundational dementia knowledge using the Dementia Knowledge Assessment Tool 2 (DKAT2), which involves 21 statements with response options of “yes,” “no,” or “don't know.” The higher the number of statements correctly answered indicates more dementia knowledge. We selected the DKAT2 because it is validated in English and Spanish and has been used in Chinese.^46^, ^47^, ^48^

We evaluated self‐efficacy to care for a person living with dementia using the Caregiver Self‐Efficacy (CSE) scale, which includes 10 statements with response options on a 1–10 rating scale, clustered into two domains: Symptom Management (five statements; 5–50 score range) and Community Support Service Use (four statements; 4–40 score range). One statement—“find ways to pay for services”—did not load into either domain. We selected the CSE because it is validated in English and has been used in Spanish.^49^, ^50^ The Chinese version of the CSE has not been used in research or validated to our knowledge; we conducted translation‐back‐translation to use this instrument.

#### 2.4.3 Level 3 measures—caregivers’ behavior changes transferred to the workplace

Since data collection relied on caregiver self‐report, we used various proxies to measure caregivers’ behavior changes to deliver person‐centered care in the workplace. We assessed caregiver's perceptions about the effectiveness of their interactions with care recipients, using five statements with response options on a five‐point scale (“strongly disagree” to “strongly agree”). Statements aimed to capture the ease of general communication, ease of discussing health‐related matters, caregivers’ feelings of being valued by their care recipients, caregivers’ sense of inclusion as active participants in the healthcare team, and caregivers’ skills to present a problem without blaming the care recipient. These questions were related to and distinct from the measurement of self‐efficacy at Level 2, which emphasized how certain or confident caregivers were in their abilities to manage their care recipient's dementia‐related symptoms and service support needs.

We also evaluated overall job satisfaction using a single‐item question—“How satisfied are you with your job in general?”—with response options on a five‐point scale (“very dissatisfied” to “very satisfied”). This single‐item question has been empirically found to be an inclusive measure of overall job satisfaction.^51^

#### 2.4.4 Level 4a measures—changes in broader healthcare practices

Since data collection relied on caregiver self‐reports, we used various proxies to measure changes in the delivery of care within health systems. This was a two‐part set of questions. The first part comprised three items with response options on a five‐point scale (“strongly disagree” to “strongly agree”). These items evaluated caregivers’ perception of healthcare providers’ reliance on them for information about their care recipients’ health needs, caregivers’ comfort level in communicating with their care recipients’ healthcare providers, and caregivers’ self‐assessed knowledge of effective communication strategies with healthcare providers to ensure their care recipients’ needs are met. The second set of questions consisted of three items with response options on a four‐point scale (“never” to “always”), measuring how often caregivers communicated with their care recipients’ healthcare providers regarding their care recipients’ health conditions, well‐being, and quality of life.

#### 2.4.5 Level 4b measures—changes in clinical outcomes and benefits for caregivers

We evaluated caregiver distress using the Caregiver Self‐Assessment Questionnaire (CSAQ), which involves 18 statements. The first 16 statements have response options of yes/no, followed by two statements with response options on a 1–10 rating scale. Chances of experiencing high distress are true if any of these criteria are met—(1) “Yes” is answered to either or both statements #4 and #11, (b) the total “Yes” response is 10 or more, or (c) statements #17 or #18 score a 6 or higher. We selected the CSAQ because it is validated in English and has been used in Spanish and Chinese.^52^, ^53^

We evaluated caregiver depression using the Patient Health Questionnaire‐2 (PHQ‐2), which involves two statements with response options on a 0–3 rating scale. The possible score range is 0‐6. A score of 3 or greater has a sensitivity for depressed mood over the past 2 weeks. We selected the PHQ‐2 because it is validated in English, Spanish, and Chinese.^54^, ^55^, ^56^

Caregiver health and well‐being are often neglected due to the stressors and responsibilities of their caregiving role, so we assessed caregivers’ health and well‐being by asking two questions with response options on a four‐point scale (“never” to “always”). The questions evaluated the frequency with which caregivers exercised for personal health and prepared meals for themselves or their families that consisted of at least half fruits and vegetables.

#### 2.4.6 Level 4b measures—changes in clinical outcomes and benefits for care recipients

AAH created data files to provide a retrospective look at healthcare services used by care recipients 12 months before and after their caregiver participated in the training. The requisite 12‐month pre‐ and post‐training interval meant that records reflected a healthcare utilization date range of August 2019–July 2023, which aligned with caregivers who participated in the training intervention between September 2020 and June 2022. Data files abstracted by AAH included medical claims and encounters; the California Department of Health Care Services’ monthly eligibility files of care recipient enrollment and demographics from Medicaid applications through the County Social Services office, Health Care Options, or Ombudsman's office; and group care enrollment files. Utilization data included five service types: emergency room visits, inpatient hospitalizations, skilled nursing facility admission/acute rehabilitation, office visits, or telehealth visits. AAH transferred the final data file to the University of California, San Francisco (UCSF) in April 2024 for analysis.

### 2.5 Data analysis

CCA and AAH de‐identified caregiver and care recipient data before they transferred it to the UCSF research team for analysis. We conducted quantitative analyses using Stata/SE 17. We used descriptive statistics to summarize demographic data, job skills, knowledge, awareness of AAH resources, training satisfaction, and health and well‐being. To assess changes in dementia knowledge, self‐efficacy, and depression, we performed repeated measures univariate analysis of variance (ANOVA), with post‐hoc comparisons adjusted using the Bonferroni test. We applied a significance level of *α* = 0.017 to determine statistical significance. To assess changes in distress, we used a generalized estimating equation model. We standardized utilization rates to 1000 members per year to allow for comparisons across different population sizes and healthcare enrollment durations. We conducted paired t‐tests for the significance of pre‐ and post‐training healthcare utilization for each type of service.

## 3 RESULTS

Among 597 caregivers who completed the training, 206 had missing or incomplete survey data (e.g., incorrectly entered participant identification number) that we could not link among pre‐, post‐, and 3‐month follow‐up time periods. To account for this, we conducted a complete case analysis in which only non‐missing data were included in each pre‐, post‐, and 3‐month follow‐up analysis, resulting in 391 complete caregiver cases. Further, we linked complete caregiver cases to available care recipient healthcare service use data. Due to the requisite 12‐month pre‐ and post‐training interval, we received utilization data for care recipients whose caregivers participated in the training between September 2020 and June 2022. We did not receive utilization data for care recipients whose caregivers participated in the intervention between August 2022 and December 2023 due to the conclusion of the grant period before the requisite 12‐month post‐training interval had elapsed, as well as administrative time lags before medical claims and encounters data could become available. The resulting analytic sample includes 209 caregiver and care recipient dyads (**Figure** **3**). From this analytic sample, 61% of caregivers took the training in English, 25% in Chinese (Cantonese), and 14% in Spanish.

### 3.1 Characteristics of IHSS caregivers and care recipients

Table 1 displays the demographic characteristics of caregivers and care recipients in this study. Among caregivers, the sample consisted of women (87%), with a mean age of 50 years (range: 20–75 years). Ethnically and racially, 62% were non‐Hispanic/Latino, 21% were Hispanic/Latino, 38% were Asian/Asian American, and 25% were Black/African American individuals. Most caregivers were family members (66%), with children or children‐in‐law being the most common familial relationship (46%). Most caregivers lived separately from their care recipients (56%), had been providing care for 5 years or less (67%), and were paid to work under 40 h per week (77%).

**TABLE 1:** IHSS caregiver and care recipient characteristics (*N* = 209).

Demographic	Caregivers *n* (%)	Care Recipient *n* (%)
Age, in years (± SD, min‐max)	50.6 (± 11.2, 20–75)	72.7 (± 11.7, 50‐96)
Diagnosis, before—after intervention		
Dementia		44 (21%) 53 (25%)
Alzheimer's disease		19 (9%) 29 (14%)
Ethnicity		
Hispanic or Latino	44 (21%)	34 (17%)
Not Hispanic or Latino	130 (62%)	173 (83%)
Prefer not to answer or no answer	35 (17%)	1 (0%)
Race		
American Indian or Alaska Native	2 (1%)	0 (0%)
Asian or Asian American	80 (38%)	77 (37%)
Black or African American	52 (25%)	51 (24%)
Native Hawaiian or Other Pacific Islander	3 (1%)	13 (6%)
White or Caucasian	13 (6%)	12 (6%)
Not listed	33 (16%)	20 (10%)
Selected more than one race	6 (3%)	0 (0%)
Prefer not to answer or no answer	20 (10%)	36 (17%)
Language preference		
English	127 (61%)	99 (47%)
Spanish	29 (14%)	30 (14%)
Chinese	53 (25%)	56 (27%)
Gender identity		
Women	181 (87%)	159 (76%)
Men	23 (11%)	50 (24%)
Prefer not to answer or no answer	2 (1%)	0 (0%)
Sexual orientation		
Straight/heterosexual	146 (70%)	
Gay/lesbian/bisexual	10 (5%)	
Prefer not to answer or no answer	16 (8%)	
Marital status		
Married/partnered	120 (57%)	
Divorced	17 (8%)	
Separated	6 (3%)	
Widowed	6 (3%)	
Never married	49 (23%)	
Prefer not to answer or no answer	11 (5%)	
Education		
Grade school (K‐12 or GED)	80 (38%)	
Some college, no degree	41 (20%)	
Trade, technical, or vocational school	22 (11%)	
Associate degree	20 (10%)	
Bachelor's degree	31 (15%)	
Advanced degree	9 (4%)	
Prefer not to answer or no answer	6 (3%)	
Household size		
Mean (± SD)	3.5 (± 1.55)	
Annual household income		
$49,999 or less	112 (54%)	
$50,000–$99,999	52 (25%)	
$100,000 or more	2 (1%)	
Prefer not to answer or no answer	43 (21%)	
Total number of IHSS work experience years		
5 years or less	106 (51%)	
6–10 years	60 (29%)	
More than 10 years	42 (20%)	
Prefer not to answer or no answer	1 (0%)	
Relationship to care recipient		
Spouse or partner	17 (8%)	
Daughter/son or daughter/son‐in‐law	95 (46%)	
Another relative	25 (12%)	
Friend or neighbor	23 (11%)	
Non‐family independent provider	33 (16%)	
Other	16 (8%)	
Prefer not to answer or no answer	0 (0%)	
Living arrangement		
Not with care recipient	116 (56%)	
With care recipient	92 (44%)	
Prefer not to answer or no answer	1 (0%)	
No. of years working for care recipient		
5 years or less	139 (67%)	
6–10 years	49 (23%)	
More than 10 years	21 (10%)	
Prefer not to answer or no answer	0 (0%)	
No. of paid work hours/week for care recipient		
39 h or less	160 (77%)	
40–65 h	34 (16%)	
66–90 h	13 (6%)	
Prefer not to answer or no answer	2 (1%)	
Power of attorney for care recipient		
Yes	9 (4%)	
No	200 (96%)	
Prefer not to answer or no answer	0 (0%)	

Among care recipients, the sample comprised of women (76%), with a mean age of 72 years (range: 50–96 years). Ethnically and racially, 83% were non‐Hispanic/Latino, 17% were Hispanic/Latino, 37% were Asian/Asian American, and 24% were Black/African American individuals. Linguistic diversity was distinct, with English as the most prevalent (47%), followed by Chinese (27%), and Spanish (14%). Before the intervention, 21% of care recipients had a dementia diagnosis and 9% had an Alzheimer's diagnosis.

### 3.2 Level 1 outcomes—caregivers’ reactions to the training

Pre‐training data revealed that 89% of caregivers had no prior dementia training. Post‐training data indicated that 98% of caregivers were satisfied or very satisfied with the training. The majority agreed or strongly agreed that the training was beneficial (97%), they learned new caregiving skills (97%), communication with their care recipients improved (96%), the instructor made them feel comfortable (99%), and the instructor answered questions effectively (98%). Regarding format, 51% of caregivers found the 3‐h weekly sessions ideal for optimal learning, while 20% preferred 2.5 hours, 23% preferred 2 h, and 6% preferred 1.5 hours. Notably, 95% would recommend the training to others.

Caregivers’ access to essential resources for participating in online training improved between pre‐ and post‐training, including consistent access to a stable Internet connection (78% to 92%), access to a computer equipped with a camera (71% to 80%), availability of a smartphone or tablet with a camera (89% to 94%), and access to a quiet space for learning (79% to 84%). These improvements in technological and environmental resources indicate enhanced readiness for online learning among caregivers, potentially contributing to the effectiveness of the intervention.

### 3.3 Level 2 outcomes—caregivers’ acquisition of knowledge, skills, and self‐efficacy

Caregivers consistently reported high levels of caregiving skills, with between 87% and 92% affirming they have the skills needed to perform their job well, could meet all their care recipient's needs, and felt comfortable asking others for help when needed at pre‐training, post‐training, and 3‐month follow‐up. Results revealed slightly mixed changes in caregivers’ IHSS‐related knowledge. Correct understanding of emergency care for falls with bruising and swelling decreased (90% to 67% to 63%) across the study period, while knowledge improved in understanding that caregivers cannot give medication directly unless under the supervision of a health professional (33% to 83% to 89%), not to double a missed medication dose (4% to 98% to 96%), and that preventing falls is important to avoid hospitalizations (87% to 95% to 96%). Caregivers’ knowledge of accessing various AAH resources also improved for urgent care (70% to 92% to 95%), member services (75% to 96% to 92%), the nurse advice line (69% to 92% to 92%), and behavioral health services (49% to 82% to 82%) across the study period.

Results revealed improvements in overall dementia knowledge, with mean DKAT2 scores rising from 11.8 to 15.5 at post‐training and 15.2 at 3‐month follow‐up, all maintaining statistical significance compared to pre‐training (*p* < 0.001). Significant improvements (*p* < 0.001) were noted in 15 DKAT2 statements at post‐training and 14 at 3‐month follow‐up. These findings indicate the training effectively enhanced caregivers’ knowledge of dementia care, with improvements enduring at 3‐month follow‐up for most areas (Table 2).

**TABLE 2:** Dementia knowledge assessment tool (*N* = 209).

	Correctly answered statements			% Change		*p*‐Value	
	Pre n (%)	Post n (%)	FU n (%)	Pre‐post	Pre‐FU	Pre‐post	Pre‐FU
DKAT2 mean score (SD)	11.8 (± 3.4)	15.5 (± 2.4)	15.2 (± 2.9)	31%	29%	<0.001	<0.001
Individual statements							
1. Dementia occurs because of changes in the brain.	193 (92%)	202 (97%)	204 (98%)	5%	6%	0.08	0.021
2. Brain changes causing dementia are often progressive.	168 (80%)	197 (94%)	197 (94%)	17%	17%	<0.001	<0.001
3. Alzheimer's disease is the main cause of dementia.	121 (58%)	167 (80%)	167 (80%)	38%	38%	<0.001	<0.001
4. Blood vessel disease (like vascular disease) can also cause dementia.	98 (47%)	148 (71%)	144 (69%)	51%	47%	<0.001	<0.001
5. Confusion in an older person is almost always due to dementia.	73 (35%)	97 (46%)	86 (41%)	33%	18%	0.004	0.246
6. Only older adults develop dementia.	134 (64%)	153 (73%)	146 (70%)	14%	9%	0.033	0.323
7. Knowing the likely causes of dementia can help to predict its progression.	21 (10%)	39 (19%)	21 (13%)	86%	29%	0.01	0.981
8. Incontinence always occurs in the early stages of dementia.	72 (34%)	141 (67%)	130 (62%)	96%	81%	<0.001	<0.001
9. Dementia is likely to limit life expectancy (decrease or shorten how long the person will live).	89 (43%)	134 (64%)	134 (64%)	51%	51%	<0.001	<0.001
10. When a person has late‐stage dementia, families can help others to understand that person's needs.	188 (90%)	188 (90%)	189 (90%)	0%	1%	0.99	0.99
11. People who have dementia may develop problems with visual perception (understanding or recognizing what they see).	162 (78%)	185 (89%)	189 (90%)	14%	17%	<0.001	<0.001
12. Sudden increases in confusion are characteristic of dementia.	12 (6%)	31 (15%)	30 (14%)	158%	150%	0.003	0.006
13. Uncharacteristic distressing behaviors may occur in people who have dementia (e.g., aggressive behavior in a gentle person).	164 (78%)	200 (96%)	203 (97%)	22%	24%	<0.001	<0.001
14. Difficulty swallowing occurs in late‐stage dementia.	103 (49%)	181 (87%)	184 (88%)	76%	79%	<0.001	<0.001
15. Movement (e.g., walking, moving in a bed or chair) is limited in late‐stage dementia.	129 (62%)	178 (85%)	177 (85%)	38%	37%	<0.001	<0.001
16. Changing the environment (e.g., putting on a CD, opening or closing the blinds) will make no difference to a person who has dementia.	89 (43%)	149 (71%)	148 (71%)	67%	66%	<0.001	<0.001
17. When a person who has dementia is distressed, it may help to talk to them about their feelings.	160 (77%)	185 (89%)	185 (89%)	16%	16%	<0.001	<0.001
18. It is important to always correct a person who has dementia when they are confused.	82 (39%)	170 (81%)	156 (75%)	107%	90%	<0.001	<0.001
19. A person who has dementia can often be supported to make choices (e.g., what clothes to wear).	165 (79%)	190 (91%)	193 (92%)	15%	17%	<0.001	<0.001
20. It is impossible to tell if a person who is in the later stages of dementia is in pain.	67 (32%)	111 (53%)	91 (44%)	66%	36%	<0.001	0.02
21. Exercise can sometimes be of benefit to people who have dementia.	172 (82%)	203 (97%)	204 (98%)	18%	19%	<0.001	<0.001

*Note*: Statistically significant change, *p*‐value < 0.001.

Abbreviation: DKAT2, Dementia Knowledge Assessment Tool Version Two.

In terms of self‐efficacy, both the Symptom Management and Community Support Services domains demonstrated significant improvements between pre‐ and post‐training as well as between pre‐training and 3‐month follow‐up (*p* < 0.001). In the Symptom Management Domain, mean scores increased from 35.6 to 41.7 at post‐training and 41.8 at 3‐month follow‐up. The Community Support Services Use domain saw mean scores rise from 27 to 32.2 at post‐training and 31.6 at 3‐month follow‐up. Although self‐efficacy in “find ways to pay for services” did not load into either domain, mean scores increased by 43% at post‐training, maintaining this improvement at 3‐month follow‐up (*p* < 0.001). These findings indicate the training effectively improved caregivers’ self‐efficacy, with improvements sustaining at 3‐month follow‐up for most areas (Table 3).

**TABLE 3:** Caregiver self‐efficacy scale (*N* = 209).

	Mean (SD)			% Change		*p*‐Value	
Parameter	Pre	Post	FU	Pre‐post	Pre‐FU	Pre‐post	Pre‐FU
Symptom management domain	35.6 (± 10.6)	42.7 (± 7.1)	41.8 (± 8.2)	20%	17%	<0.001	<0.001
Community support service use domain	27.0 (± 9.5)	32.2 (± 6.5)	31.6 (± 7.5)	20%	17%	<0.001	<0.001
Individual statements: How certain are you right now that you can:							
1. Handle any problems your care recipient has, like memory loss, wandering, or behavior problems. (symptom management domain)	6.70 (± 2.55)	8.57 (± 1.45)	8.35 (± 1.71)	28%	25%	<0.001	<0.001
2. Handle any problems that might come up in the future with your care recipient's care. (symptom management domain)	7.05 (± 2.45)	8.54 (± 1.53)	8.42 (± 1.67)	21%	19%	<0.001	<0.001
3. Deal with the frustrations of caring for your care recipient. (symptom management domain)	7.27 (± 2.39)	8.61 (± 1.41)	8.34 (± 1.74)	18%	15%	<0.001	<0.001
4. Do something to keep your care recipient as independent as possible. (symptom management domain)	7.21 (± 2.34)	8.53 (± 1.58)	8.33 (± 1.88)	18%	15%	<0.001	<0.001
5. Get answers to all your questions about your care recipient's problems. (symptom management domain)	7.36 (± 2.29)	8.57 (± 1.42)	8.45 (± 1.74)	17%	15%	<0.001	<0.001
6. Care for your care recipient without help from organizations or agencies that provide services. (community support service use domain)	6.31 (± 2.69)	7.24 (± 2.42)	7.33 (± 2.36)	15%	16%	<0.001	<0.001
7. Find organizations or agencies in the community that provide services to help your care recipient. (community support service use domain)	6.87 (± 2.67)	8.37 (± 1.88)	8.10 (± 2.07)	22%	18%	<0.001	<0.001
8. Get answers to all your questions about these services. (community support service use domain)	6.92 (± 2.61)	8.39 (± 1.72)	8.20 (± 1.92)	21%	18%	<0.001	<0.001
9. Arrange for these services yourself. (community support service use domain)	6.89 (± 2.70)	8.25 (± 1.82)	8.00 (± 2.07)	20%	16%	<0.001	<0.001
10. Find ways to pay for these services.	4.82 (± 2.87)	6.87 (± 2.53)	6.88 (± 2.66)	43%	43%	<0.001	<0.001

*Note*: Symptom management self‐efficacy domain includes statements 1–5 and has a possible score range of 5–50. Community support service use self‐efficacy domain includes statements 6–9 and has a possible score range of 4–40. Statistically significant change, *p*‐value < 0.001.

Abbreviation: FU, follow‐up.

### 3.4 Level 3 outcomes—caregivers’ behavior changes transferred to the workplace

Proxy measures of caregivers’ behavior changes transferred to the workplace and job satisfaction remained relatively stable between pre‐training and 3‐month follow‐up. Specifically, a high percentage of caregivers reported ease of communication with care recipients (89% to 84%), ease of talking to care recipients about health (85% to 81%), feeling valued by care recipients (91% to 89%), believe they are participating members of the healthcare team (90% to 86%), and can present problems without blaming or judging their care recipients when conflicts arise (87% to 85%). The percentage of caregivers who were satisfied or very satisfied with their job stayed high (85% to 80%) between pre‐training and 3‐month follow‐up.

### 3.5 Level 4a outcomes—changes in healthcare practices

Proxy measures of healthcare practice changes remained relatively stable between pre‐training and 3‐month follow‐up. A high percentage of caregivers identified themselves as collaborators with other healthcare providers (e.g., doctors, nurses, psychologists, physician assistants, care managers, care coordinators, and social workers) (78% to 76%), felt comfortable communicating with healthcare providers (88% to 81%), and knew how to communicate effectively with healthcare providers (86% to 85%). Approximately two‐thirds of caregivers felt that healthcare providers relied on them for information on how to address the health needs of their care recipients (65% to 69%) and frequently communicated with healthcare providers about their care recipient's health conditions (66% to 61%). About half frequently communicated with healthcare providers about their care recipient's well‐being and quality of life (54% to 55% for both).

### 3.6 Level 4b outcomes—changes in clinical outcomes and benefits for caregivers

The CSAQ indicated high distress in 49% of caregivers at pre‐training, 56% at post‐training, and 45% at 3‐month follow‐up. A significant change (*p* = 0.03) was found for CSAQ results between post‐training and 3‐month follow‐up. The PHQ‐2 indicated depressed mood in 4% of caregivers at pre‐training and 6% at both post‐training and 3‐month follow‐up. No significant differences were found in the PHQ‐2 results.

The frequency of exercising three times a week remained relatively stable (47% to 46%), as did the frequency of preparing meals with at least half fruits and vegetables (81% to 78%) between pre‐training and 3‐month follow‐up.

### 3.7 Level 4b outcomes—changes in clinical outcomes and benefits for care recipients

**FIGURE 4:**
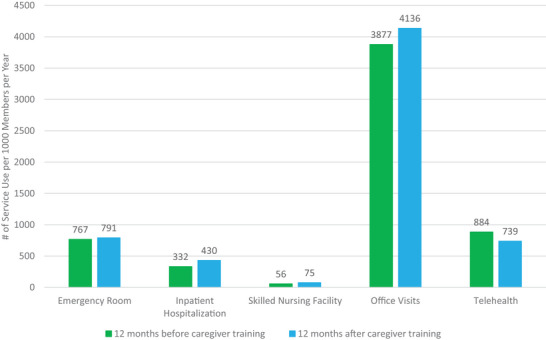
Care recipients’ healthcare utilization rates before and after caregiver training.

**FIGURE 5:**
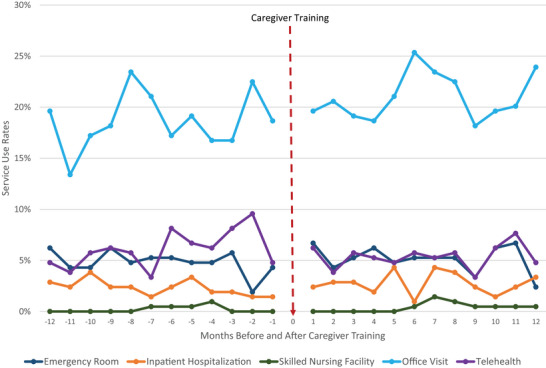
Care recipients’ monthly service use before and after caregiver training (*N* = 209).

Consumers’ healthcare utilization rates were standardized to per 1000 members per year for the 12 months before and after caregiver training (Figure 4). Emergency room visits increased by 3% (767 before, 791 after), while inpatient hospitalizations increased by 30% (332 before, 430 after). Figure 5 illustrates the percentage of consumers who utilized each service at least once monthly in the 12 months before and after caregiver training, with the intervention marked as time “0.” Before training, 1.9%–6.2% of care recipients had at least one emergency room visit, averaging 2.5 (± 5.1) visits among those with at least one, whereas after training, 2.4%–6.7% had at least one emergency room visit, averaging 2.4 (± 3.8) visits. Before training, 1.4%–3.8% of care recipients had at least one inpatient hospitalization, averaging 1.9 (± 2.5) hospitalizations among those with at least one, whereas after training, 1.0%–4.3% had at least one hospitalization, averaging 2.5 (± 3.1) hospitalizations. Changes in service use were not statistically significant.

Lastly, during the 12 months after caregiver training, the percentage of care recipients with a dementia diagnosis increased to 25%, Alzheimer's diagnosis increased to 14%, and 10% of care recipients died.

## 4 DISCUSSION

The IHSS+ ADRD Training Project significantly improved caregivers’ dementia knowledge and self‐efficacy, supporting our first hypothesis that the intervention would enhance these primary outcomes. These results align with prior research on the importance of specialized dementia training for home care workers.^26^, ^27^, ^28^, ^29^, ^30^, ^31^, ^32^, ^41^ However, while the intervention was effective in improving some level 2 outcomes, it did not significantly impact other indicators, such as caregivers’ confidence in their professional skills, perceived competence in addressing care recipient needs, and ability to ask for social support. This lack of impact is likely due to a ceiling effect, as these scores were already high at pre‐training and remained stable at post‐training and 3‐month follow‐up. These findings indicate that additional strategies are required to further strengthen caregivers’ skills and perceived competence in areas where they already feel proficient. Future evaluations should employ measures sensitive enough to detect subtle changes in these outcomes.

Caregivers who completed the intervention reported high satisfaction, indicating the training met their expectations. Open‐ended survey responses revealed the training fostered peer connections, social engagement, and supportive social networks. Caregivers, often working in isolation, valued sharing experiences and exchanging tips, with many expressing a desire to maintain contact after the program. One caregiver noted, “From being in this training, I got to meet many people in the field with similar experiences who made me feel less alone in the situation I'm in.”

Contrary to our second hypothesis, the training did not notably reduce caregiver distress and depression. Although our results indicated a significant decrease in distress at 3‐month follow‐up, 45% of caregivers still reported experiencing high distress. Sources of caregiver stress and burden are well‐documented,^12^, ^13^, ^57^ with some sources likely exacerbated by the long duration of the COVID‐19 pandemic.^10^ Mitigating these stressors is challenging,^27^ and only one of the 10 training modules focused on reducing caregiver stress, specifically. While about half of caregivers reported elevated distress, fewer than 6% reported depression, suggesting the need for targeted interventions. Open‐ended survey responses indicated that caregivers understood the importance of their well‐being within the caregiving dyad relationship. One caregiver shared, “The impact [of the training] was teaching me my feelings are valid, and my health is important. I need to care for myself just like I would care for my [care recipient].”

Contrary to our third hypothesis, we did not find significant differences in emergency room visits and hospitalizations after caregiver training. Changes in healthcare needs due to declining cognition and increased acuity among care recipients, potentially exacerbated by the COVID‐19 pandemic, along with the shift to telehealth, delays in preventive care, and heightened awareness of urgent health issues from training, likely contributed to more frequent urgent in‐person medical visits or admissions. These distal outcomes should be interpreted cautiously to avoid ecological fallacies about person‐level service use based on group‐level service use, as healthcare usage was not adjusted for ADRD diagnosis or other factors, and the pandemic's impact remains uncertain.

Although 66% of the caregivers in this study were family members, 11% were friends or neighbors, and 16% were non‐family independent providers. This training has applications for family and non‐family caregivers and may be appropriate for caregivers of individuals with preclinical or subjective cognitive impairment. The core of our assertion lies in the ability of the training to equip caregivers with the knowledge and skills necessary to recognize early signs of cognitive decline, potentially helping them document changes, facilitate timely discussions with healthcare providers, and advocate for their care recipients who might otherwise be overlooked. Delayed screening, detection, and underdiagnosis of probable ADRD are major clinical concerns.^3^, ^43^, ^44^ Approximately a quarter of care recipients in our sample had a dementia diagnosis, with even fewer diagnosed with Alzheimer's. Increasing caregivers’ dementia knowledge and self‐efficacy through training could potentially lead to earlier dementia screening and detection among care recipients, improving access to therapies, resources, and support services, and extending care recipients’ ability to age well at home. The training also emphasizes preventive measures, such as creating safe environments and promoting healthy behaviors, which are crucial for managing early dementia symptoms and potentially slowing the progression of the disease.

### 4.1 Limitations

This study has several limitations, including selection biases from participant self‐selection, technological readiness, and monetary incentives. Participants opting in for eligibility screening may have been more aware of their care recipient's cognition changes, potentially diminishing the intervention's effect. Conversely, most caregivers were not caring for a care recipient with an ADRD diagnosis, which may have made them less attentive to dementia‐related behaviors, or their enrollment was motivated by monetary incentives.

Self‐reported measures for caregiver outcomes may have introduced recall and desirability biases, and the COVID‐19 pandemic may have confounded certain outcomes. The study's focus on a single California county limits generalizability to other regions or demographics. The absence of a control group constrained our ability to attribute changes solely to the intervention, and attrition issues compromised data integrity. While our design choices allowed for the execution of the study in a real‐world setting within available means, it inherently restricts the strength of causal inferences that can be drawn from the results.

Most caregivers had multiple years of experience, which may have reduced the training intervention's impact compared to those with less experience. Multivariate analyses exploring subgroup effects based on caregiver or care recipient characteristics were not conducted.

We did not receive healthcare utilization data for care recipients whose caregivers participated in the training between August 2022 and December 2023, due to the grant period ending before the required 12‐month post‐training interval and administrative delays in data availability. Consequently, long‐term utilization patterns could not be assessed. Lastly, we did not account for healthcare services reimbursed by Medicare for dual‐eligible Medicare and Medicaid care recipients.

### 4.2 Future directions

Future research should strengthen ongoing policy efforts that support the continued professional development and well‐being of home care workers. For instance, incorporating a control group with intent‐to‐treat analyses could strengthen the ability to attribute outcomes directly to the intervention. Comparative analyses of different training modalities could optimize intervention design. Multivariate analyses could help identify potential subgroup effects and inform more customized approaches to caregiver education and support. Longitudinal studies with extended follow‐up periods could provide insights into the durability of training effects and long‐term impacts on caregivers and care recipients.^21^

The impact of caregiver training on care recipients’ healthcare utilization remains difficult to measure.^21^ Individuals who qualify for IHSS are often high‐acuity and assessed by social workers as needing caregiver support to live independently, which, without IHSS funding, might necessitate placement in an institution. Detailed explorations of individual cases could help to understand whether and for whom increased use represents appropriate service use or indicates areas needing further care recipient and caregiver support. While healthcare providers should recognize caregivers’ enhanced roles and integrate them into the care team and care planning processes, a gap persists between these recommendations and the marginalization of caregivers within the healthcare system.^58^, ^59^, ^60^ For instance, the Centers for Medicare and Medicaid Services launched the Guiding an Improved Dementia Experience (GUIDE) Model in July 2024 to enhance caregiver support and resources by providing a care navigator, respite, and education on caring for a loved one with dementia.^61^ It remains to be seen whether the GUIDE Model can address care recipients’ health concerns that caregivers encounter, as well as other issues that lead to caregiver distress. Despite the potential rewards of dementia caregiving, issues such as caregiver burden, anxiety, and loneliness are often overlooked in the broader healthcare context, necessitating future research into clinical and policy interventions, such as educating healthcare providers on integrating caregivers into care teams to enhance support and reduce distress.^6^, ^11^, ^12^, ^13^

Additionally, this training could be implemented outside of California, as much of the dementia and caregiving content is not IHSS‐specific. California's robust IHSS program provided us with the opportunity for a larger sample size, offering valuable insights that could inform the implementation and evaluation of future ADRD training programs across the country. Adapting and replicating the curriculum for home care workers nationwide aligns with broader initiatives to create dementia‐friendly and age‐friendly ecosystems.^62^, ^63^, ^64^, ^65^, ^66^ Including more non‐family home care workers or independent providers and expanding the training's geographical scope could enhance the generalizability of findings and inform adaptation strategies for different contexts. This approach supports California's goal of creating one million high‐quality caregiving jobs by 2030 as part of the Master Plan for Aging^67^ and national efforts to empower and improve recruitment, training, and retention of direct care workers and family caregivers, in line with initiatives of the Direct Care Workforce Strategies Center and National Plan to Address Alzheimer's Disease.^33^, ^68^, ^69^

Lastly, enhanced long‐term care policies could better support the home care workforce, such as by establishing higher pay rates from Medicaid‐funded personal care programs to support a living wage and providing basic employee benefits, including professional development opportunities, paid leave, retirement plans, and health insurance.^59^, ^70^ Future efforts should invest in implementing and evaluating comprehensive, sustainable training programs to assess their impact on home care workforce well‐being and effectiveness, and care recipient outcomes in dementia care.

## AUTHOR CONTRIBUTIONS

Jarmin Yeh was responsible for funding acquisition, project supervision, management, and drafting the original manuscript. Andrea Garcia, Juliana Mata‐Pacheco, Juvenal Mauricio, Moraima Castaneda, and Corinne Eldridge were responsible for delivering the training intervention. Jarmin Yeh, Matthew Beld, Brittney Pond, Melinda Neri, and Suzanna Martinez were responsible for data analysis. All authors contributed to the project implementation, acquisition of data, interpretation of data, and critical revision of the manuscript for significant intellectual content and provided final approval of the submitted version.

## CONFLICT OF INTEREST STATEMENT

The authors declare no conflicts of interest. Author disclosures are available in the Supplementary Information.

## CONSENT STATEMENT

The Institutional Review Board of the University of California, San Francisco, did not consider this to be human subjects research, and consent was not necessary.

## Supporting information



Supporting Information

Supporting Information
