# Supplementary material for: Competency‐based training boosts dementia knowledge and skills in home care workers
Source: Alzheimers Dement. 2025 Jun 19;21(6):e70323. doi: 10.1002/alz.70323 (PMC12179336; doi:10.1002/alz.70323)
Supplement: Supplementary file 1 — Supporting Information [file ALZ-21-e70323-s002.pdf]

Supplemental Materials. Samples of the IHSS+ ADRD Training Project weekly modules and learning modalities

<table>
<tr><th>Weekly Modules</th><th>Types of Learning Modalities</th></tr>
<tr><td>

Module 1: Roles and Responsibilities of the Caregiver

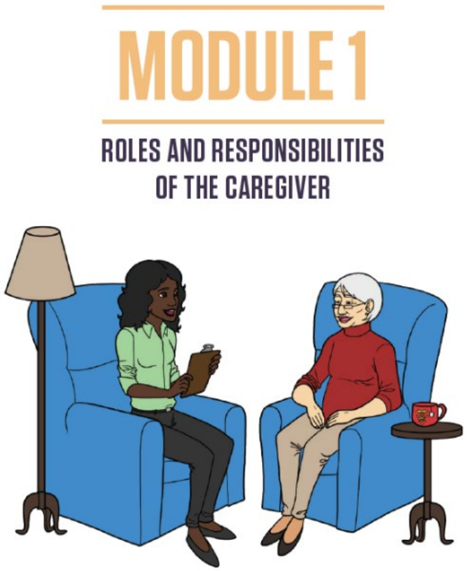

This module will orient you to the training series. It introduces the roles and responsibilities of IHSS caregivers. These enhanced roles, along with the skills of the IHSS caregiver, are key to providing high-quality care for care recipients with Alzheimer's disease and dementia.

Learning Objectives:

- Identify the roles and responsibilities of caregivers.
- Describe the five enhanced roles of the caregiver on the care team: *monitor, coach, communicator, navigator,* and *care aide.*
- Describe the four key skills that support the enhanced roles: *observe, monitor, document,* and *report.*
- Identify important health plan information from a medical card.
- Appropriately document and report changes in or concerns about care recipient behavior.

</td><td>

Example of Interactive Group Exercises:

SECTION 1.4

Care Scenarios

Instructions: In your group, read the scenario you have been assigned. Answer the questions on the slide. As you answer the questions, refer to the Documentation Form and consider your responsibilities to observe, monitor, document, and report.

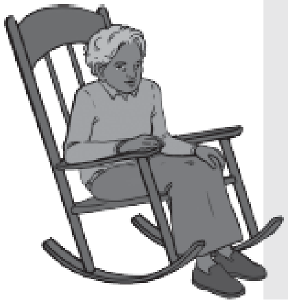

SCENARIO A: Teresa has recently been diagnosed with high blood pressure. You are a new caregiver for her. Teresa is 80 years old and has Alzheimer's disease. For most personal needs she is fairly independent, except for preparing her meals. During her last medical visit, her primary care physician directed her to decrease the sodium content in her diet.

</td></tr>
</table>

Module 2: Person-Centered Care, Communication Skills, Care Recipient and Caregiver Rights

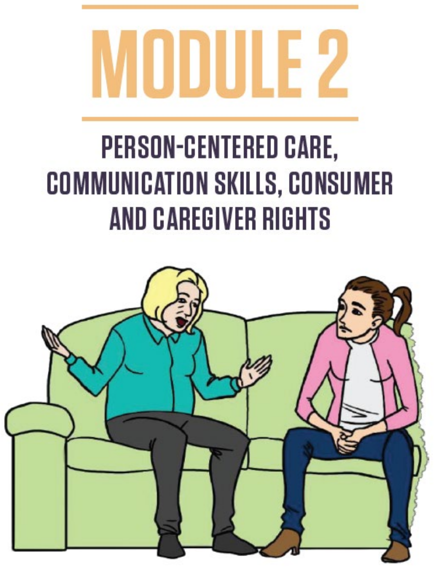

In this module, you will practice communication skills, including how to effectively discuss a problem with a care recipient. Caregivers will also learn about person-centered care and be introduced to the rights of caregivers and care recipients.

Learning Objectives:

- Describe person-centered care and discuss its importance when caring for care recipients.
- Describe and demonstrate effective skills for communicating with care recipients.
- Confidently discuss a problem with a care recipient.
- Demonstrate strategies for resolving conflict and talking about sensitive issues with care recipients and members of their care teams.
- Identify the rights of caregivers and care recipients.

Example of Worksheets:

Body language

Instructions
Think about what your body language is really "saying" to others. Discuss with your partner each of the examples of body language listed in the table below. Next to each example, write the possible meanings or interpretations others might have for that body language.

Body Language	Possible Meanings
Frowning	
Having a slumped posture	
Avoiding eye contact	
Looking at cell phone while someone is talking	
Folding arms across chest	
Tapping fingers	
Looking away	

Module 3: Recognizing Alzheimer's and Dementia

MODULE 3

RECOGNIZING ALZHEIMER'S AND DEMENTIA

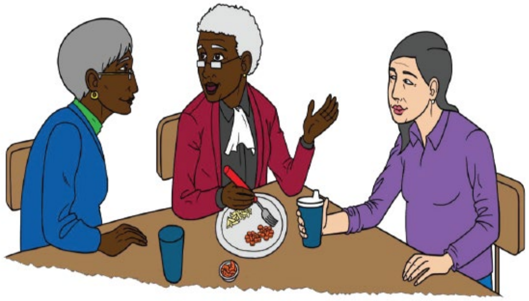

In this module, you will learn to recognize (though not diagnose) symptoms of Alzheimer's disease and dementia, which differ from the signs of normal aging. You will learn what symptoms and stages caregivers may expect to find as they care for care recipients with these conditions, and you will discuss how to apply the skills of observing, monitoring, documenting, and reporting in these scenarios.

Learning Objectives:

- Define Alzheimer's disease.
- Define dementia.
- Identify the signs of Alzheimer's disease and dementia, distinguishing between these and the normal signs of aging.
- Describe common stages of Alzheimer's disease.
- Describe how to appropriately use the four key skills – *observe, monitor, document,* and *report* – while caring for care recipients with these conditions.

Example of Worksheets:

SECTION 3.3

Understanding the Difference between Alzheimer's Disease and Dementia

Write down the differences between Alzheimer's and dementia:

____

____

____

____

____

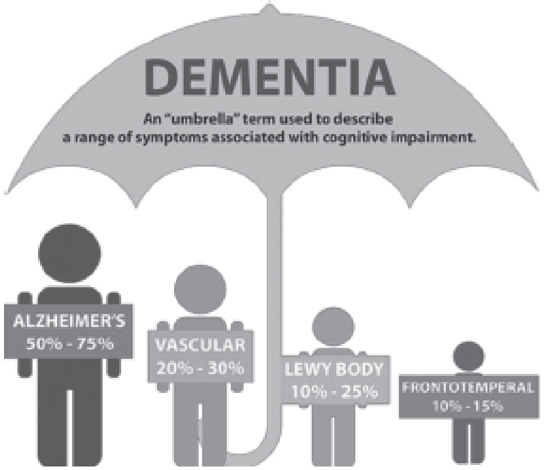

- Dementia is an umbrella term.
- Alzheimer's is a disease and a type of dementia.
- Alzheimer's is the most common type of dementia.

» Dementia is a group of symptoms.
The number one cause of dementia is Alzheimer's Disease.

Module 4: Common Behaviors and Sundowning

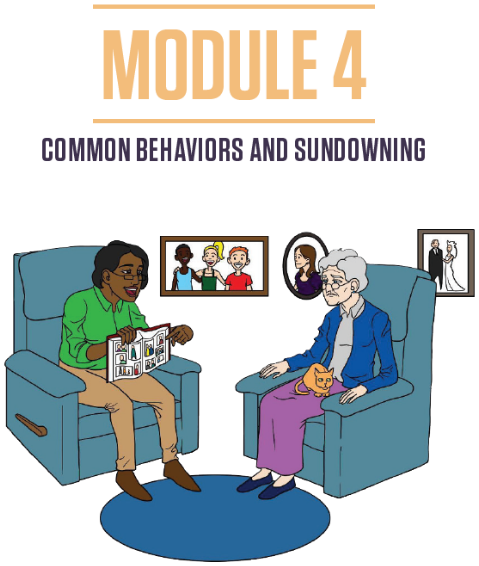

In this module, you will learn about common behaviors associated with Alzheimer's disease and dementia. The focus will be on challenging and repetitive behaviors, such as repetitive questioning, and practicing techniques for helping care recipients when they occur. You will also learn about techniques for assisting care recipients with agitation and anxiety, as well as providing appropriate care. Delirium, what it is, and how caregivers should respond, will also be reviewed. Finally, participants will discuss sundowning, a common symptom of Alzheimer's disease and dementia.

Learning Objectives:

- Demonstrate strategies to help care recipients with repetitive behaviors and questions.
- Describe strategies for responding to challenging behaviors.
- Demonstrate techniques to lessen agitation and anxiety.
- Recognize delirium and possible causes.
- Identify symptoms of sundowning.
- State important considerations in the care of a care recipient with sundowning.

Example of Interactive Videos:

Video — Repetitive Behaviors

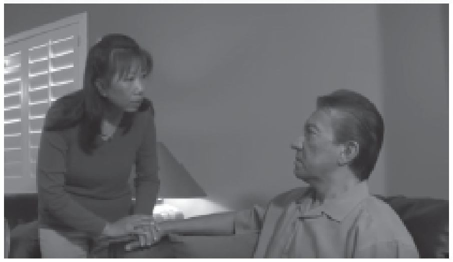

http://bit.ly/2S6CfXY

1. What tips for addressing repetitive behaviors were suggested in the video?

____

____

____

2. Are you able to use these tips with the consumer you care for? If so, how?

____

____

____

Module 5: Sleep Changes, Hallucinations, Personal and Home Safety, Wandering

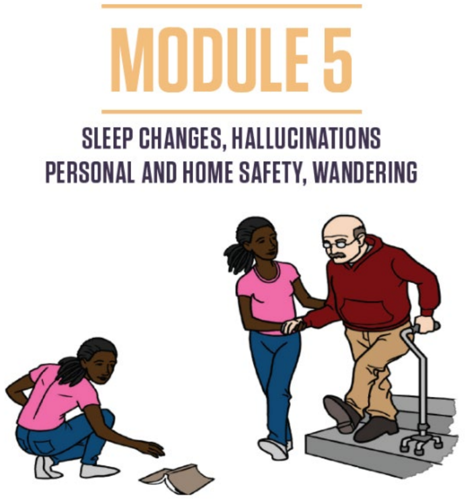

In this module, you will first learn about sleep hallucinations. By understanding the principles of good sleep hygiene, you will be able to encourage better sleep. You will also learn to identify when the care recipient is experiencing hallucinations and provide appropriate care.

Another important topic discussed in this module is safety in the home for both caregivers and care recipients. Caregivers have an increased risk for workplace musculoskeletal injuries because of the ongoing physical support they give to the care recipient. You will learn how to use good body mechanics to avoid injury. Care recipients with Alzheimer's disease and dementia are at risk of falls, so strategies to prevent falls will be introduced. Another safety issue concerns care recipient wandering. Wandering is a common symptom of dementia. Strategies to prevent wandering will be discussed.

Learning Objectives:

- Describe principles of good sleep hygiene and identify strategies to help care recipients with Alzheimer's disease or dementia sleep better.
- Identify signs of hallucinations and describe key elements of appropriate care for care recipients who experience these symptoms.
- Identify strategies to prevent hallucinations.
- Describe specific techniques in body mechanics to prevent injury on the job.

Example of Take-Home Assignments:

SECTION 5.7

Take-Home Assignment

Name ______________________________

- Review all the materials you have read today. This week take the *Home Safety Checklist*, on pages 28-29, with you to the consumer's home. Each part of this tool corresponds to a different area in the consumer's home. Check these areas in the consumer's home and fill out the chart below with things that may be fall hazards.
- You may be able to "fix" some of the hazards you find. For example, you can remove any soap build-up you find in the tub and secure area rugs that might be tripping hazards. If you can, take action. Note what you did below.

Potential fall hazards in the home	Action you took, if any	If you cannot take action, who will you report this to?

• Describe why appropriate and functional durable medical equipment (DME) is an important part of home safety.<br>• Describe specific actions to prevent the care recipient with Alzheimer's disease or dementia from falling.<br>• Identify ways to prevent wandering for the care recipient with Alzheimer's disease or dementia.	

Module 6: Assigning with Personal Hygiene

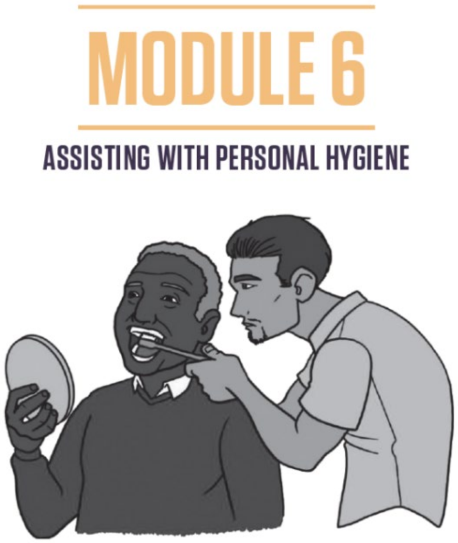

In this module, you will learn how to assist care recipients with maintaining personal hygiene, such as oral and denture care, bathing, dressing, and using the toilet. You will explore special considerations for assisting a care recipient with Alzheimer's disease and dementia with these tasks and learn how to effectively address situations in which care recipients refuse to do something.

Learning Objectives:

- Assist a care recipient with proper oral and denture care.
- Assist a care recipient with bathing and dressing.
- Assist a care recipient with using the toilet.
- Describe special considerations for assisting care recipients with Alzheimer's disease and dementia that may be necessary to maintain their personal hygiene.
- Demonstrate strategies to use when a care recipient with dementia refuses to engage in a personal hygiene activity, such as bathing or oral care.

Example of Competency Checks:

SECTION 6.8

Assisting a Consumer to Use the Toilet

Reflecting on what was discussed in Module 3, what are three possible reasons a consumer with Alzheimer's disease or dementia would have trouble using the toilet?

1. ________________________________

2. ________________________________

3. ________________________________

Module 7: Adult CPR Training

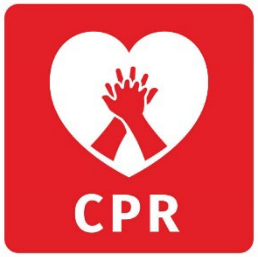

In this module, you will become CPR-certified through a blended learning format of online lectures and in-person skills assessments.

Learning Objectives:

- Recognize and respond appropriately to cardiac, breathing, and first aid emergencies to know to give immediate care to a suddenly injured or ill care recipient until more advanced medical personnel arrive and take over.

Example of Blended Online and In-Person Skills Assessment:

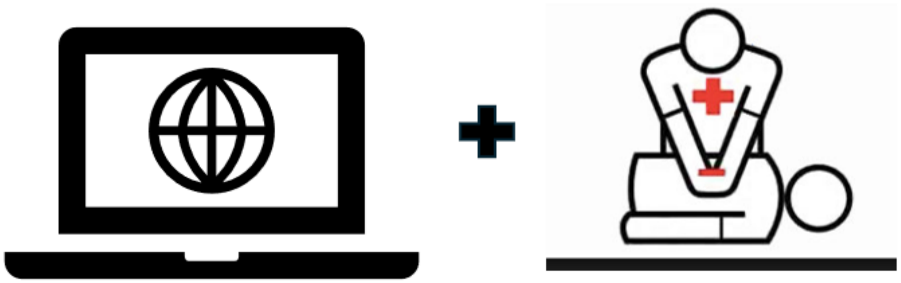

Module 8:
Nutrition and Physical Activity

MODULE 8

NUTRITION AND PHYSICAL ACTIVITY

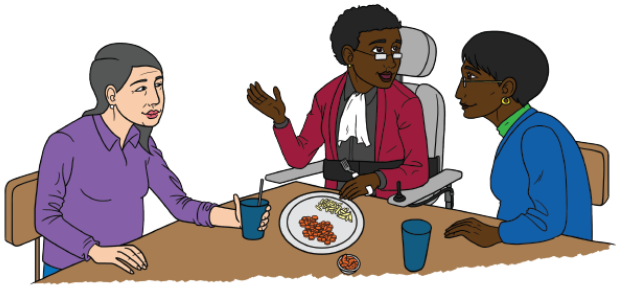

In this module, you will learn the basics of nutrition, healthy and modified diets, as well as the importance of physical activity in a care recipient's daily routine. Caregivers will learn about assistive devices that can help a care recipient to eat. They will also be introduced to chronic conditions linked with Alzheimer's disease.

Learning Objectives:

- Describe the basics of good nutrition.
- Explain the importance of respecting the care recipient's cultural and personal food preferences, as well as all medically necessary dietary restrictions and needs.
- Describe common, simple, and modified diets.
- Demonstrate how to assist a care recipient with eating.
- Identify recommendations for physical activity for care recipients.
- Understand the connection between diabetes and Alzheimer's disease.
- Identify symptoms and treatment options for depression experienced by care recipients with Alzheimer's disease and dementia.

Example of Worksheets:

The label should read:

**Fat**	Less than 5 grams of total fat
**Sugar**	Less than 10g of sugar
**Salt**	Less than 300mg of sodium

Healthy or unhealthy?

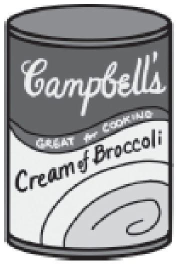

Nutrition Facts
Serv. Size 1/2 cup (12 ml) condensed soup
Servings about 2.5

Calories 90
Fat Cal 50
* Percent Daily Values (DV) are based on a 2,000 calorie diet

Amount/serving	%DV*	Amount/serving	%DV*
Total Fat 5g	8%	Sodium 790mg	33%
Sat. Fat 1g	5%	Potassium 95mg	3%
Trans Fat 0g		Total Carb. 9g	3%
Polyunsat. Fat 1g		Fiber 1g	4%
Monounsat. Fat 2g		Sugars 3g	
Cholest. 5mg	2%	Protein 1g	
Vitamin A 6% • Vitamin C 0% • Calcium 2% • Iron 2% • Zinc 70%			

Fat: ______________________________

Sugar: ______________________________

Salt: ______________________________

Healthy or unhealthy? ______________________________

Module 9: Medications – Urgent Care Versus Emergency Care

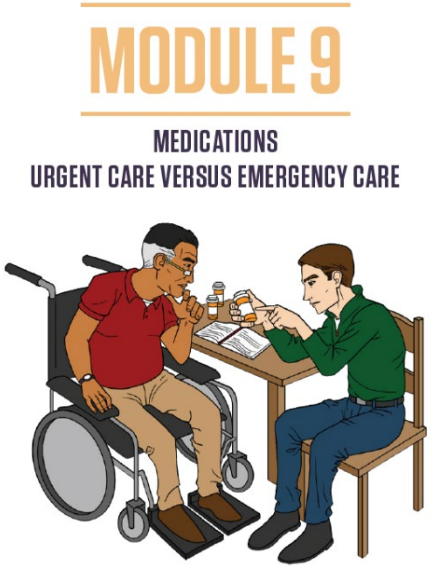

In this module, you will learn various safety considerations for assisting with medications. In addition, you will be introduced to techniques and tools for medication management, including strategies that can help when care recipients with Alzheimer's disease or dementia refuse to take their medication. You will also learn criteria to use in making the decision between choosing urgent care or emergency care for the care recipient. You will learn how to prepare, what to bring, and what questions to expect from the healthcare providers.

Learning Objectives:

- Read a prescription label properly for key information.
- Explain ways to prevent medication errors.
- Communicate effectively with care recipients to assist them to take medicine properly.
- Describe how to help prevent medication-related falls.
- Identify strategies to assist with medication refusals in care recipients with Alzheimer's disease or dementia.
- Use criteria to decide between the need for urgent care versus emergency care.
- Identify the items you need to bring with you to urgent or emergency care when assisting a care recipient with Alzheimer's disease or dementia, in order to be fully prepared.

Example of Worksheets:

Instructions: Read the prescription label below. Find and circle the Five Rights.

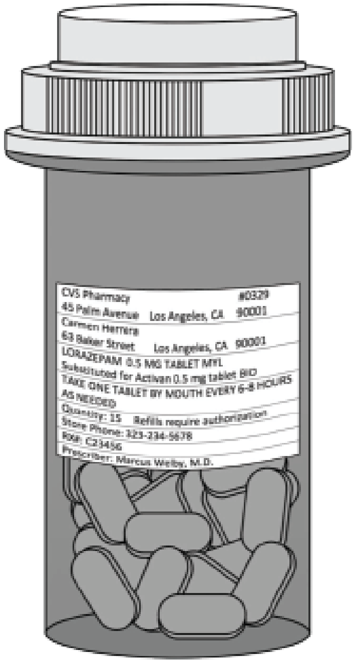

CVS Pharmacy
#0329<br>45 Palm Avenue<br>Los Angeles, CA 90001
Carmen Herrera<br>63 Baker Street<br>Los Angeles, CA 90001
LORAZEPAM 0.5 MG TABLET<br>Substituted for Ativan 0.5 mg tablet
TAKE ONE TABLET BY MOUTH<br>EVERY 6-8 HOURS AS NEEDED<br>FOR ANXIETY AND AGITATION
Quantity: 15<br>Refills require authorization
Store Phone: 323-234-5678
RX#: C23456
Prescriber: Marcus Welby, M.D.

Module 10: Reducing Caregiver Stress & Competency Checks

In this module, you will learn strategies for recognizing burnout and reducing caregiver stress. You will then complete five competency checks.

Learning objectives:

- Identify ways to reduce caregiver stress and burnout.
- Demonstrate at least one stress reduction technique.
- Compete competency checks.

MODULE 10

REDUCING CAREGIVER STRESS
COMPETENCY CHECKS

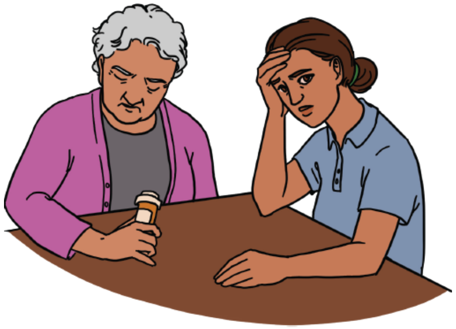

Example of Role Playing:

SECTION 10.2

Comprehensive Assessments

1. Role-Play: Responding to Dementia-Related Behavior

Instructions: How can you deal with this situation in a person-centered way?

> SCENARIO: Albert is a 76-year-old consumer with dementia. You are his care provider. Later in the afternoon, Albert often starts to become disoriented and can be physically aggressive, especially when it is time for you to assist him to take his eye drops. You try to be gentle and patient, but it's difficult because you have to get close to him to assist with the drops and you are afraid he might hit you. One afternoon, Albert grabs a large, heavy flashlight from the kitchen counter and holds it over his head, threatening you.

Consider the comments below when preparing your role-play. You can also use the material from Module 4 in your workbook to prepare your role-play.

- Albert is behaving aggressively. Try to determine why this is happening by using a person-centered approach.
- What strategies you can use to calm Albert?
- What strategies you can use to calm yourself and keep from getting hurt?
